# Porous Si-SiO_2_ based UV Microcavities

**DOI:** 10.1038/s41598-020-59001-7

**Published:** 2020-02-10

**Authors:** María R. Jimenéz-Vivanco, Godofredo García, Jesús Carrillo, Vivechana Agarwal, Tomás Díaz-Becerril, Rafael Doti, Jocelyn Faubert, J. E. Lugo

**Affiliations:** 1 Centro de Investigación en Dispositivos Semiconductores, ICUAP, BUAP, Ciudad Universitaria., Puebla, Puebla 72570 México; 2 CIICAP- Universidad Autónoma del Estado de Morelos, Av. Universidad 1001, Col Chamilpa, Cuernavaca, Morelos, México; 3 Faubert Lab, École d’optométrie, Université de Montréal, Montréal, 3744 Jean Brillant, Montréal, H3T 1P1 Québec Canada

**Keywords:** Synthesis and processing, Photonic crystals

## Abstract

Obtaining silicon-based photonic-structures in the ultraviolet range would expand the wavelength bandwidth of silicon technology, where it is normally forbidden. Herein, we fabricated porous silicon microcavities by electrochemical etching of alternating high and low refraction index layers; and were carefully subjected to two stages of dry oxidation at 350 °C for 30 minutes and 900 °C, with different oxidation times. In this way, we obtained oxidized porous silicon that induces a shift of a localized mode in the ultraviolet region. The presence of Si-O-Si bonds was made clear by FTIR absorbance spectra. High-quality oxidized microcavities were shown by SEM, where their mechanical stability was clearly visible. We used an effective medium model to predict the refractive index and optical properties of the microcavities. The model can use either two or three components (Si, SiO_2_, and air). The latter predicts that the microcavities are made almost completely of SiO_2_, implying less photon losses in the structure. The theoretical photonic-bandgap structure and localized photonic mode location showed that the experimental spectral peaks within the UV photonic bandgap are indeed localized modes. These results support that our oxidation process is very advantageous to obtain complex photonic structures in the UV region.

## Introduction

Porous Silicon (PS) is a material used in the manufacture of one-dimensional Photonic Crystals: Bragg Reflectors (BRs)^1,2^, microcavities (MCs)^3,4^, Rugate Filters^5,6^, and waveguides^7^. These can be formed by interchanging a sequence of different dielectric layers, each one with different refractive index. The refractive indexes are obtained by applying different current pulses, which change the porosity in each layer. Some optoelectronic devices such as sensors^8,9^, Light Emitting Diodes^10^ and photodetectors^11^ have been developed as well. Unfortunately, natural oxidation of the PS pores walls occurs, and it is contaminated by impurities when in contact with air^12,13^; therefore, it is an unstable material. In the past years, the thermal oxidation of Crystalline Silicon (Si) was arduously investigated mainly focusing on the influence of different oxidation characteristics such as the major role of pre-oxidation approaches in the strategy of thermal growth of high grade oxides on Si^14^. Therefore, stabilizing the PS optical parameters is solved by inducing its oxidation. This oxidation process improves PS transparency at short wavelengths of the VIS spectrum^15^ because silicon dioxide (SiO_2_) is a transparent material with low polarizability^16^. The porous texture in p^+^ and p^−^ Si substrates is very sensitive to heat treatment; even at low temperatures (around 400 °C) a thickening of the texture is observed which reduces the surface area and the reactivity of PS to oxidation; this effect increases with temperature^17^. The thermal oxidation process does not alter the morphology of the porous layers; only the pore size decreases after oxidation; however, the pore surface density is conserved^18^. Due to the low difference in thermal expansion coefficients between Si and SiO_2_, the oxide formation inhibits the PS skeleton relaxation^19^. V. Agarwal proposed a method that modifies the photonic bandgap (PBG) of PS structures by introducing sub-mirrors coupled with MCs to explore three different wavelength bandwidths from ultraviolet (UV) to near infrared (NIR). In order to stabilize these mirrors, they were partially oxidized with dry oxidation^20^. Gelloz used High-Pressure Water Vapor Annealing (HWA) for the stabilization of BRs obtained at low anodization temperatures (−20 °C) using p-type Si; HWA was conducted at pressures from 1.3 to 2.6 MPa, at 260 °C and for three hours; this method improves the transparency of PS layers with an efficient response in the UV region due to a high oxidation of the PS structures^21^. F. Morales has manufactured BRs at room temperature. To stabilize the optical parameters of BRs in the UV range dry oxidation is performed^22^. BRs based on Oxidized Porous Silicon (OPS) and TiO_2_ were manufactured by Christian R. Ocier. In the first stage, PS BRs with a stopband at 530 nm were fabricated and then were thermally oxidized. After oxidation, the stopband shifted to 440 nm. In the second stage, OPS BRs were infiltrated with TiO_2_, and the stopband red-shifted to 492 nm; at maximum infilling, with TiO_2_, the stopband had a transmission of 2% (at 620 nm)^23^. M. Ghulinyan and C. J. Oton reported MCs centered in the infrared region where the PS is almost transparent; absorption losses play a much less important role than other loss mechanisms such as light scattering by the pores and the interfaces between layers^12,24^. Light scattering is one of the drawbacks for photon transmission in PS due to the disorder typically occurring within it^25^. Specifically, photon losses occurring within the near infrared interval are dominantly connected to Rayleigh scattering^26^. Irrespectively, scattering losses in PS waveguides can be diminished by oxidation. For instance, propagation losses in the visible and near infrared spectra were measured but when the PS waveguides were oxidized the losses decreased^27^. In another study Vorozov *et al*. have achieved a 75% reduction of the scattering losses in PS waveguides after oxidation^28^.

Taking into consideration the aforementioned Si and PS pre-oxidation and oxidation concepts, hereunder we present the manufacturing of porous silicon MCs with optical response in the UV range, which is a two-fold process. First, we fabricated porous silicon microcavities consisting of two Bragg reflectors with a defect layer between them with optical response in the blue range. The MCs had a localized mode inside their PBG. Second, the MCs were subjected to two stages of dry oxidation. The first stage is a pre-oxidation at low-temperature of 350 °C, which is necessary to stabilize the silicon structure, to avoid the coalescence of the pores during further treatments at higher temperature^29^. The second stage was performed at high oxidation temperature of 900 °C. This oxidation process transforms almost completely the PS MCs into porous SiO_2_ MCs, as it was indicated by our three-component effective medium approximation, the high visible light transparency of the oxidized MCs and the presence of prominent Fourier-Transform Infrared Spectroscopy (FTIR) Si-O-Si peaks. Hence, this oxidation transformation induces an UV shift of the MCs localized mode, and a decrease of the optical losses within the MCs. In order to assure that the porous multilayers structure after oxidation was preserved, we used Scanning Electron Microscopy (SEM). Furthermore, we theoretically fitted the experimental transmission and reflection spectra before and after dry oxidation; and the theoretical bandgap structure and localized mode location were calculated. The experimental localized mode was found inside the forbidden PBG and close to the theoretical localized mode prediction. The MCs optical losses were qualitatively assessed via the absorbance spectrum, whose amplitude decreased more than 50% in the UV light range and almost disappeared within the visible light after oxidation. We also followed the changes of the localized mode transmission peak, whose amplitude and bandwidth are modified by optical losses. A modified Breit-Wigner equation was used to get the dispersion in the localized mode due to absorption and scattering losses^25,30^; from this equation we estimated photon loss rates, which includes both types of losses, Rayleigh scattering and light absorption, whereby the lifetime of photons and photon loss can be defined at the localized mode wavelength.

Finally, we demonstrate here that it is possible to obtain highly transparent MCs within the UV range thanks to the dry oxidation carefully carried out in two stages. This result opens up the possibility of novel PS based photonic devices.

## Results and Discussion

### Porous silicon microcavities geometric characterization

**Figure 1:**
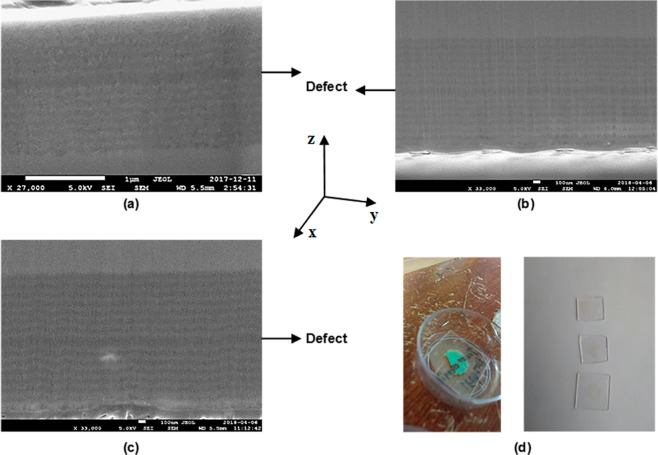
(**a**) Cross-section SEM of a microcavity on a p^+^ Si substrate with 31 layers without oxide. (**b**) Cross-section SEM of an oxidized microcavity (OM), first at 350 °C for 30 minutes, and then the temperature was increased to 900 °C for one hour. (**c**) SEM image of the cross-section of an OM first at 350 °C for 30 minutes and then at 900 °C for 2 hours. (**d**-left) Unoxidized microcavity on a quartz substrate. (**d**-right) Three highly transparent OMs. All SEM images show a defect layer between two BRs (black arrows). The layers with low porosity can be observed in light gray, and layers with high porosity are displayed in dark gray. The microcavities have a total of 31 layers, where the z-axe is taken along of the normal direction to the layers.

Figure 1 shows the high-resolution scanning electron microscopy (SEM) images of three MCs on a p^+^ Si substrate. Alternating quarter-wave layers with high refractive index (*n*_*H*_) and low refractive index (*n*_*L*_) were fabricated to create two BRs with a defect layer (refractive index *n*_*d*_) between them. The Bragg Reflectors had a PBG in the blue band, approximately between 420 nm to 560 nm. The layers with low porosity can be observed in light gray, and layers with high porosity are displayed in dark gray (Fig. 1). There is a defect layer between two BRs, which has twice the thickness of the high porosity layer. Based on the gravimetric method^20^ the porosity and thickness for each monolayer of unoxidized PS are known. The microcavity porosities we obtained without oxide (Fig. 1a) were 39% and 74%, and thicknesses *d*_*H*_ = 30.2 nm and *d*_*L*_ = 64.6 nm; hence it has a total thickness of roughly 1.5 μm while from the SEM image we estimated a total thickness of approximately 1.6 μm, where individual layers have thickness of *d*_*H*_ = 35 nm and *d*_*L*_ = 68.75 nm. These values are very close to the thicknesses obtained by gravimetry; there is a slight difference of 4.8 nm between both estimations.

In Fig. 1b,c, are shown SEM measurements of two oxidized microcavities (OMs). They were subjected to two different stages of dry oxidation, the first microcavity (Fig. 1b) was oxidized at 350 °C for 30 minutes and then at 900 °C for one hour; the second microcavity was oxidized at 350 °C for 30 minutes and then the temperature was increased to 900 °C for two hours. SEM measurements showed that the total thickness of the first OM (Fig. 1b) is approximately 1.92 μm with thicknesses *d*_*H*_ = 48 nm and *d*_*L*_ = 75 nm, while the total thickness of the second microcavity (Fig. 1b) is approximately 2.083 μm with thicknesses *d*_*H*_ = 50 nm and *d*_*L*_ = 81.25 nm. Another way to estimate individual layer thickness within an OM is using the Transfer Matrix Method. In this method, the experimental reflection and transmission spectra are fitted with their theoretical counterparts. Thicknesses were found for each layer within each OM using the Transfer Matrix Method. The values were *d*_*H*_ = 47.42 nm and *d*_*L*_ = 78.12 nm. These values correspond to the first OM (Fig. 1b), which from this method should have a total thickness of 1.96 μm. The second microcavity has a total thickness of 2.017 μm and individual thicknesses of *d*_*H*_ = 49.57 nm and *d*_*L*_ = 79.57 nm. These OMs values obtained by SEM and theoretical values showed a small difference between them.

### Complex refractive index of porous silicon and oxidized porous silicon

In order to calculate refractive indices and layer porosities, we applied the Effective Medium Theory (EMT) of Maxwell-Garnett for two components: Si and air^31^. This equation allows us to find the theoretical values of the complex effective refractive index as well as the air and Si volumetric fractions of each PS layer. The refractive index of each layer is a function of its porosity and wavelength. The theoretical values obtained for porosity, Si fraction, and complex refractive index of 8 MCs are shown in Table 1.

**Table 1:** Theoretical values of porosity, complex refractive index and Si fraction of 8 different MCs in the blue range (at the localized mode wavelength).

Microcavity	Porosity (%)	Complex refractive index	*f*_*Si*_ (%)
MC1	40	${n}_{H}\,=\,2.6449\,-\,0.039223i$	60
	57	${n}_{L}\,=\,2.0858\,-\,0.025241i$	43
MC2	39	${n}_{H}\,=\,2.668\,-\,0.041632i$	61
	56	${n}_{L}\,=\,2.109\,-\,0.026951i$	44
MC3	39	${n}_{H}\,=\,2.6711\,-\,0.042617i$	61
	57	${n}_{L}\,=\,2.0792\,-\,0.026798i$	43
MC4	39	${n}_{H}\,=\,2.6744\,-\,0.0423i$	61
	57	${n}_{L}\,=\,2.0817\,-\,0.026598i$	43
MC5	39	${n}_{H}\,=\,2.6504\,-\,0.035886i$	61
	55	${n}_{L}\,=\,2.1267\,-\,0.023907i$	45
MC6	37	${n}_{H}\,=\,2.702\,-\,0.036619i$	63
	55	${n}_{L}\,=\,2.1192\,-\,0.023308i$	45
MC7	32	${n}_{H}\,=\,2.8815\,-\,0.03878i$	68
	55	${n}_{L}\,=\,2.1121\,-\,0.022102i$	45
MC8	39	${n}_{H}\,=\,2.6868\,-\,0.043492i$	61
	59	${n}_{L}\,=\,2.0293\,-\,0.025762i$	41

The PS-MCs after dry oxidation are no longer a mixture of Si and air; a third component (SiO_2_) is formed to get oxidized porous silicon (OPS). We used the model proposed by J. E. Lugo, for a system of three components: Si, SiO_2_ and air to obtain the porosity (*p*_*ox*_), Si fraction (*f*_*Si*_) and SiO_2_ fraction (*f*_*ox*_), and the complex refractive index of oxidized layers^31^.

Table 2 shows the theoretical values of porosity (*p*_*ox*_), complex refractive index, Si fraction (*f*_*Si*_) and SiO_2_ fraction (*f*_*ox*_) of layers having three components. The complex refractive index and porosity of OPS decrease when SiO_2_ is present in the porous layers. This decrease is attributed to the dry oxidation where a Si fraction and an air fraction are occupied by SiO_2_ after oxidation. SiO_2_ has a refractive index lower than that of Si but slightly higher than the corresponding to air.

**Table 2:** Theoretical values of porosity, complex refractive index, Si fraction and oxide fraction of 8 OMs in the UV range (at the localized mode wavelength).

Microcavity	*p*_*ox*_ (%)	Complex refractive index	*f*_*ox*_ (%)	*f*_*Si*_ (%)
MC1	4.48	${n}_{H}\,=\,1.5904\,-\,0.069623i$	90.94	4.58
	21.02	${n}_{L}\,=\,1.3749\,-\,0.0072241i$	78.49	0.49
MC2	3.85	${n}_{H}\,=\,1.5916\,-\,0.080589i$	91.2	4.95
	19.44	${n}_{L}\,=\,1.3703\,-\,0.00036949i$	80.54	0.02
MC3	3.74	${n}_{H}\,=\,1.52\,-\,0.00084728i$	91.75	4.51
	20.76	${n}_{L}\,=\,1.3509\,-\,2.2574E\,-\,06i$	79.23	0.01
MC4	3.55	${n}_{{\rm{H}}}\,=\,1.5538\,-\,0.05938{\rm{i}}$	92.77	3.68
	20.75	${n}_{L}\,=\,1.3631\,-\,3.2221E\,-\,06i$	79.25	0
MC5	2.74	${n}_{H}\,=\,1.4603\,-\,1.0681E\,-\,06i$	97.26	0
	18.16	${n}_{L}\,=\,1.3775\,-\,0.00076155i$	81.79	0.05
MC6	1.93	${n}_{H}\,=\,1.51\,-\,0.0089762i$	96.54	1.53
	18.13	${n}_{L}\,=\,1.3741\,-\,8.3445E\,-\,06i$	81.87	0
MC7	0.24	${n}_{H}\,=\,1.5303\,-\,0.0050222i$	97.42	2.34
	18.13	${n}_{L}\,=\,1.3721\,-\,3.0415E\,-\,06i$	81.87	0
MC8	2.82	${n}_{H}\,=\,1.4718\,-\,0.0063777i$	96.77	0.41
	23.78	${n}_{L}\,=\,1.3596\,-\,0.0074387i$	75.75	0.47

Table 2 shows the calculated refractive index values from OMs. The optical path is modified by the oxidation process; it is the path taken by the light as it travels from one medium to another, also known as optical thickness; it decreases due to the change of refractive index. This decrease is shown in Table 2, column three.

A study on dry oxidation of an asymmetrical BR structure based on PS with 20 periods has been reported by G. Amato. The BRs presented a blue shift caused by a decrease in the refractive index value. There the value of *n*_*L*_ indicated that the *d*_*L*_ layers are wholly oxidized while the *n*_*H*_ value indicates that some unoxidized PS remained in the *d*_*H*_ layers; this was confirmed by measurements of photoluminescence^32^.

We found similar theoretical results (Table 2) where *d*_*L*_ layers (*n*_*L*_) contain none or very small Si fractions, and in *d*_*H*_ layers (*n*_*H*_) Si fractions are small ranging from 0% to 4.95%. The complete oxidation of PS layers depends primarily on the quantity of Si in the PS layers and their pore surface area. Therefore, they are oxidized differently.

### Silicon dioxide presence within the microcavities

**Figure 2:**
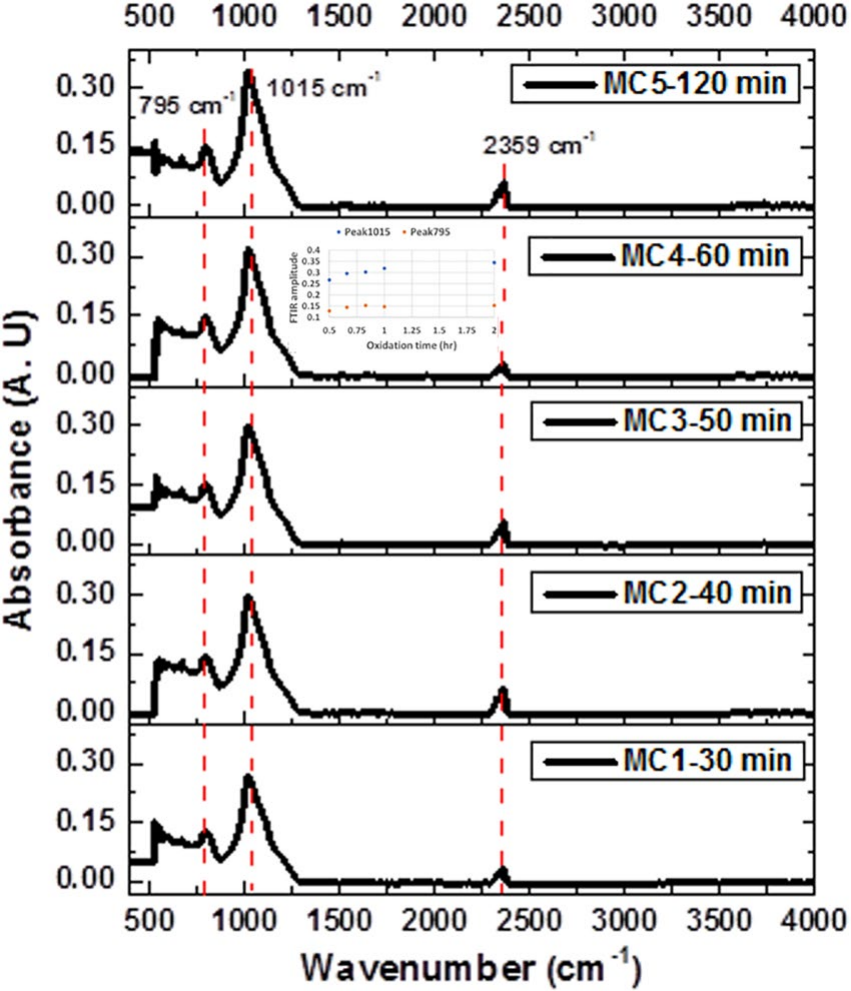
FTIR spectra of 5 MCs subjected to different oxidation times from 30 to 120 minutes. The insert shows the behavior of the FTIR amplitude vs. oxidation time for both peaks (795 cm^−1^ and 1015 cm^−1^).

Oxidation leads to the appearance of vibration bands of Si-O-Si. The spectrum in Fig. 2 shows FTIR measurements of five MCs on Si substrates subjected to different oxidation times from 30 to 120 minutes; all OMs showed the vibration mode of Si-O-Si bending at 795 cm^−1^^33,34^ and the vibration mode of Si-O-Si symmetric stretching (1015 cm^−1^)^33–36^. The peak at 2359 cm^−1^ corresponds to CO_2_ bonds^37^, which is always present in the measurements.

As the oxidation time increases from 30 to 120 minutes, there is a slight increase in both peaks amplitudes at the wavenumber representing symmetric stretching and bending vibration modes of Si-O-Si bonds (see the insert). In Table 3 is listed the exact position of the vibration type of MCs under different oxidation times.

**Table 3:** Vibration types of 5 MCs with different oxidation times from 30 to 120 minutes.

Type of vibration	The position of the main peaks
Si-O-Si bending	795 cm^−1^
Si-O-Si symmetric stretching	1015 cm^−1^
CO_2_ anti-symmetric stretching	2359 cm^−1^

These FTIR results support the fact that PS was transformed into OPS, thereby changing the optical properties of the material.

### Porous silicon microcavities on silicon substrates and quartz substrates

The Transfer Matrix Method is very well-known^38^. It was used to calculate the theoretical spectrum of transmission and reflection in MCs based on PS and OPS. Transmission and reflection spectroscopies were employed to obtain the experimental spectrum of MCs either based on PS or OPS. An UV-Vis-NIR spectrophotometer working in the wavelength range from 200 to 800 nm was used.

**Figure 3:**
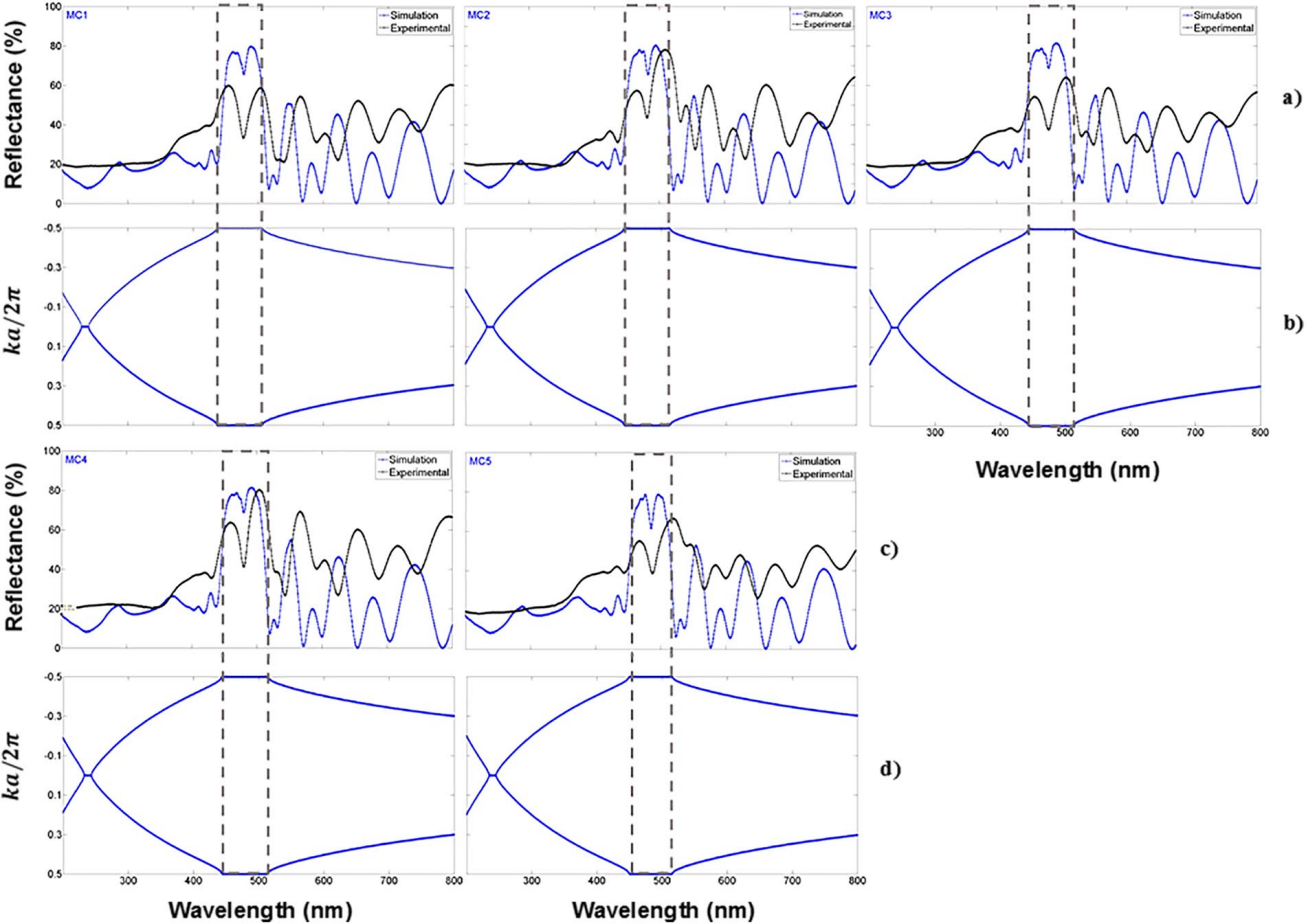
Comparison of theoretical and experimental results, (**a**,**c**) are theoretical (blue line) and experimental (black line) reflection spectra of five unoxidized MCs obtained in the VIS range; (**b**,**d**) are the photonic bandgaps (empty box) of the same five MCs. MCs have a localized mode at 480 nm. We have taken the absolute value of the complex refractive index and its average for all wavelengths for PBG calculations.

The theoretical and experimental reflection spectrum of five unoxidized MCs are shown in Fig. 3a,c. The theoretical fit was reasonable when compared with the experimental reflection spectrum. The MCs presented a localized mode^39^ having a minimum reflection peak of 40%. It is approximately positioned at a wavelength of 480 nm; Fig. 3b,d, show the PBG of the same five MCs; the localized mode of the unoxidized MCs is inside the PBG. The PBG was obtained using the dispersion relation, which can be resolved for the K (Bloch wave number) inside the first Brillouin zone. We have computed it only in one dimension and compared our theoretical calculations with the experimental results^38^.

All MCs were designed to have a localized mode at 420 nm. However, from Fig. 3 it is clear that localized modes are located around 480 nm, presenting an average shift of 60 nm towards long wavelengths (low energies).

**Figure 4:**
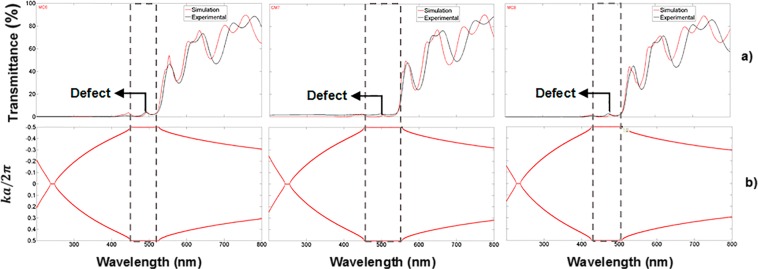
Theoretical and experimental transmission spectra and their associated photonic bandgaps of three MCs placed on quartz substrates. (**a**) Theoretical (red line) and experimental (black line) transmission spectra of three MCs obtained in the VIS range; the localized mode is located approximately at 480 nm (black arrow); (**b**) PBG structure (empty box) of the same three MCs.

Figure 4a displays experimental results of three MCs deposited on quartz substrates. The localized mode shows 5% of transmission due to photon losses. This is because porous silicon^40^ has a strong absorption in the VIS and UV ranges. Figure 3a,c, and Fig. 4a present spectra with substantial photon losses in the ultraviolet (UV); there are no apparent extended or travelling electromagnetic modes. Therefore, the absorption losses cannot be ignored in the VIS and UV ranges. In the simulation, we took into account the absorption through the complex dielectric constant (extinction coefficient and refractive index) of PS, which was introduced in the transfer matrix to obtain the transmission and reflection spectra.

Moreover, the comparison between theoretical and experimental transmission spectra is shown in Fig. 4a. We obtained a good theoretical fit. Figure 4b shows the PBGs of the same three MCs placed on quartz substrates; all localized modes are inside the PBG.

We can see a small variation of the localized mode position in the eight MCs shown in Fig. 3a,c, and 4a. It is caused by specific loss mechanisms, such as light scattering, that is, dispersion by pores, external surfaces and the interfaces between layers^12^.

These loss factors mainly depend on the size of the pores, roughness between the interfaces and the intrinsic absorption coefficient of the Si^18^. PS structures usually are oxidized to reduce optical losses. It has been reported that when obtaining PS structures by electrochemical anodization the sample surfaces are roughened; additionally, when such structures are oxidized by dry oxidation, they showed a surface roughness decrease that was a function of the oxidation temperature^18,41^.

**Figure 5:**
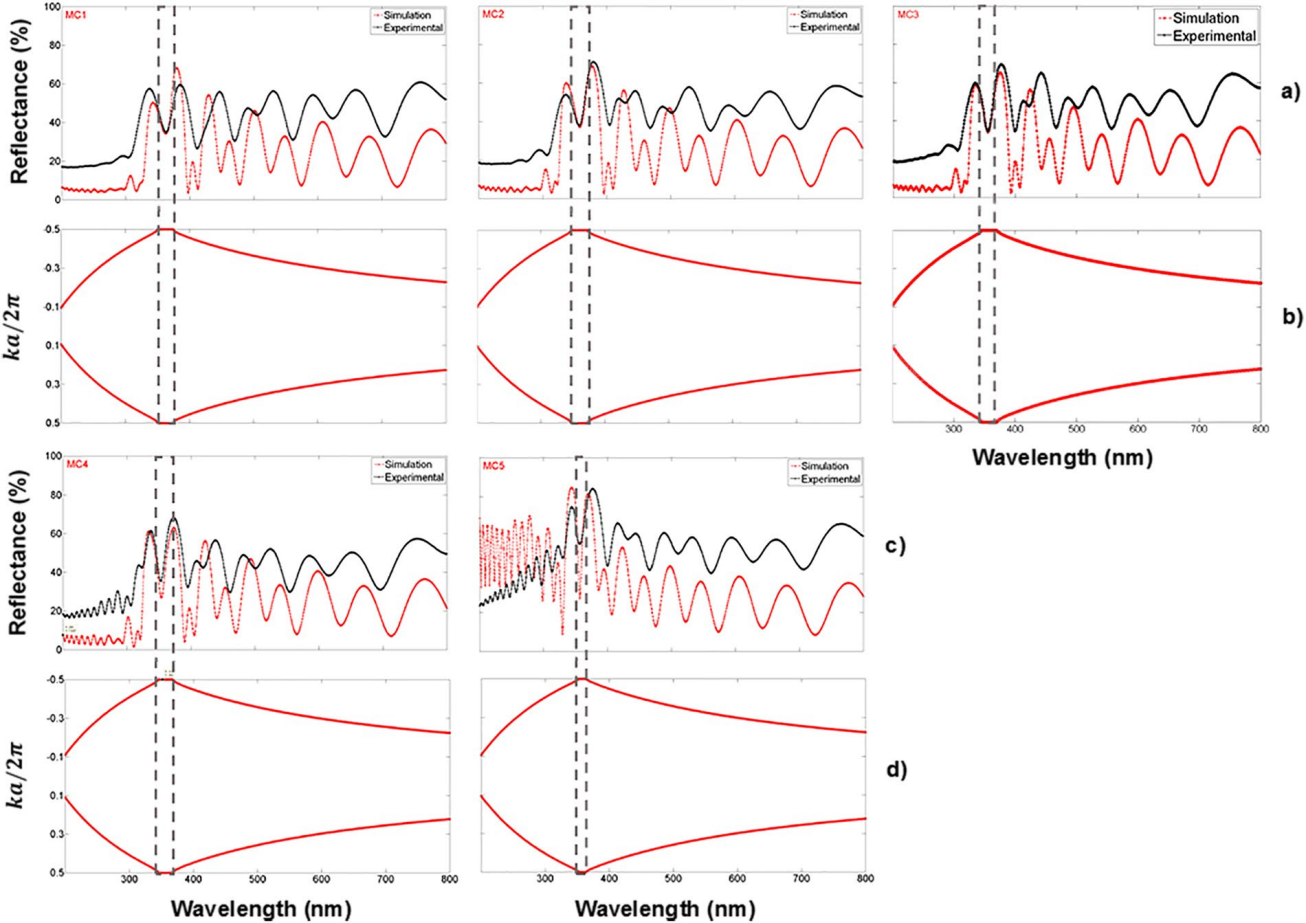
Comparison of theoretical and experimental results of five OMs in the UV range on Si substrates. (**a,c**) are the theoretical (red line) and experimental (black line) reflection spectra of five OMs, (**b,d**) are PBG structures (empty box) of the same five OMs; they have a localized mode in the UV band.

The primary purpose of subjecting MCs to dry oxidation was to obtain MCs in the UV range and thus stabilize their optical parameters such as the refractive index. In Fig. 5a,c, we show the theoretical and experimental reflection spectra of five OMs in the UV range, on a Si substrate. There is a wavelength shift of 131 nm to lower wavelengths (higher energies) of the localized mode. It is the presence of SiO_2_, within PS layers, that causes a refractive index decrease and a PS thickness increase. Therefore, the growth of SiO_2_ in PS films obeys the law valid for a Si film without pores^42^ and the combination of Si with oxygen increases the volume occupied by the solid base of the OPS film. This volume expansion occurs because the density of SiO_2_ is slightly less than that of Si^43,44^.

The MCs results displayed in Fig. 3a,c do not show any localized modes in the UV range, while the presence of localized modes is notorious in the UV-A band for the optical results displayed in Fig. 5a,c. There is also a decrease shown of the PBG bandwidth since there is less contrast between both high refractive index (*n*_*H*_) and low refractive index (*n*_*L*_) whereby the microcavity is constituted.

The amplitude of the reflection spectrum in the MCs is preserved, except for MC5, as it shows a larger amplitude in the UV range because it was oxidized for a longer time; there the presence of oxide has made the microcavity more reflective. All MCs have a localized mode in the UV range.

Table 4 shows the position of the localized mode of five MCs before and after dry oxidation. It also shows the oxidation temperature and time used to obtain OMs on Si substrates in the UV range.

**Table 4:** The position of the localized mode in five MCs on Si substrate before and after dry oxidation.

Microcavity	The localized mode in the VIS	The localized mode in the UV	shift	Dry oxidation	
				350 °C	900 °C
MC1	478 nm	363 nm	115 nm	30 min.	30 min.
MC2	481 nm	357 nm	124 nm	30 min.	40 min.
MC3	480 nm	355 nm	125 nm	30 min.	50 min.
MC4	480 nm	353 nm	127 nm	30 min.	60 min.
MC5	488 nm	357 nm	131 nm	30 min.	120 min.

**Figure 6:**
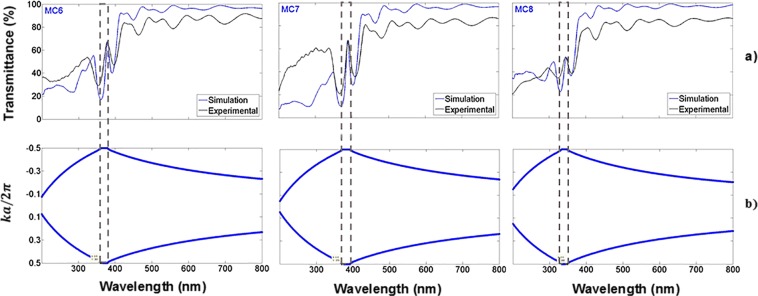
Comparison of theoretical and experimental results of three OMs in the UV range on quartz substrates. (**a**) Theoretical (blue line) and experimental (black line) transmission spectra of MCs obtained in the UV range and their localized modes are presented at 378 nm (MC6), 389 nm (MC7) and 345 nm (MC8); (**b**) PBG structure (empty box) of the same three OMs.

The transmission spectrum of three OMs placed on quartz substrates is depicted in Fig. 6a; the experimental (black line) and theoretical (blue line) spectra showed an excellent agreement between them, their PBG is shown in Fig. 6b. They are more transparent in the UV, and VIS range than MCs without oxidation and at their localized modes wavelengths show a 67% of transmission. The first microcavity (MC6) has a localized mode at a wavelength of 378 nm, where the temperature and oxidation times were 350 °C for 30 minutes, and 900 °C for 60 minutes. The microcavity MC7 has a localized mode at a wavelength of 389 nm. The temperature and oxidation time applied for microcavity MC8 were a little different than those for MC6 and MC7 microcavities. There, the first oxidation step was the same but in the second step the temperature was 900 °C for 120 minutes. Thus, we observed a more significant wavelength shift of 131 nm to lower wavelengths for the MC8 microcavity. This shift is because the microcavity has more oxide inside its layers. It showed a localized mode at a wavelength of 345 nm.

Table 5 presents the edges of the PBG of eight different MCs in the UV-VIS range; as it can be observed the PBG bandwidth in the UV range is smaller than the PBG bandwidth in the VIS range (Fig. 4). The decrease of the PBG bandwidth is due to the optical path change. The oxide inside the PS layers produced a decrease in the complex refractive index components and a thickness increase due to the lattice expansion.

**Table 5:** Edges of the PBG of eight MCs in the VIS and UV range.

Microcavity	MC1 (nm)	MC2 (nm)	MC3 (nm)	MC4 (nm)	MC5 (nm)	MC6 (nm)	MC7 (nm)	MC8 (nm)
PBG in the VIS	437–506	444–513	444–513	444–513	450–515	451–526	455–555	426–508
PBG in the UV	351–375	348–373	349–371	345–368	352–364	361–379	370–392	333–348

The effects of dry oxidation on BRs have been well studied within the VIS-NIR range^31,32,45^, where it is reported a refractive index decrease due to SiO_2_ growing inside the Si matrix. Other authors reported a mixture of OPS and titanium dioxide (TiO_2_) to form transparent BRs in the VIS range where the oxidation process eliminates optical losses across the VIS range^23^. Several studies on BRs have been made in the UV range using different Si substrates and applying two oxidation processes^20–22^. The oxidation was used for stabilization of the refractive index; the authors did not compare between experimental and theoretical results, they only supported their results with the experimental reflection spectrum confirming a decrease of light absorption, and by keeping the same amplitude reflection level in the VIS and UV range. We also obtained BRs within the UV range in this work. Our experimental and theoretical results were compared, and we found similar results (see supporting information).

Several studies of free-standing MCs and coupled MCs in the NIR region have been reported because Si is considered transparent to these wavelengths^12,13,46^; the localized mode slightly blue shifted over time as a result of the microcavity aging. A difference in the response of localized modes can be expected due to the doping inhomogeneity of the wafers as well^12^. If a reliable MC theoretical model is desired absorption losses at short wavelengths have to be taken into account, because such losses usually increases in that range.

A study on OM in the NIR band has been reported; there the effect of absorption of PS was not considered in the simulation because the extinction coefficient is very small. However, the experimental reflection spectrum with its theoretical reflection spectrum did not show a good agreement. MCs were oxidized at different temperature for 5 minutes and a wavelength shift to low wavelengths was observed^47^.

**Figure 7:**
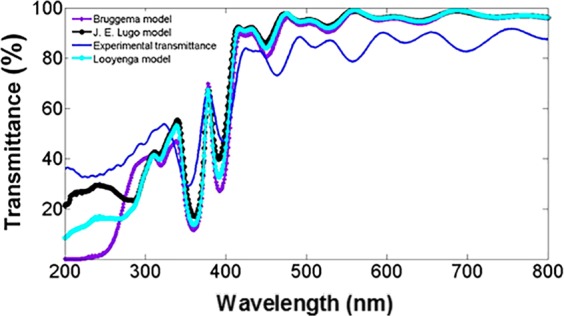
Comparison of the experimental transmission spectrum (blue line) and its theoretical counterpart for a microcavity. Three different effective medium approximations were used to model the transmission spectrum, Bruggeman (violet line), Lugo (black line) and Looyenga (light blue line).

We compared one MC experimental transmission spectrum with its theoretical counterpart obtained with three EMT approximations, namely Bruggeman, Lugo, and Looyenga (Fig. 7). Here we considered three mediums because the MCs are composed of Si, SiO_2_, and air. The results showed that the model proposed by J. E. Lugo (black line) fitted the experimental (blue line) transmission spectrum of the MC6 microcavity in the whole explored wavelength interval much better.

The other two models predicted more absorption in the UV range; that is, Bruggeman and Looyenga overestimated the amount of Si in the OPS layers, but in the whole VIS-NIR range, the three effective medium approximations predicted similar results between the experimental and theoretical transmission spectrum.

In the PBG simulation and the theoretical MCs transmission spectrum calculation on quartz substrates, we considered incident light as perpendicular to the multilayers plane. For the PBG simulation and the theoretical MCs reflection spectrum calculation on Si substrates, the incident light had an angle of 20 degrees off the perpendicular to the multilayers plane. Our theoretical calculations of bandgap structures, transmission and reflection spectra approximated the experimental results well.

### Defect mode within a microcavity in the visible and ultraviolet range

**Figure 8:**
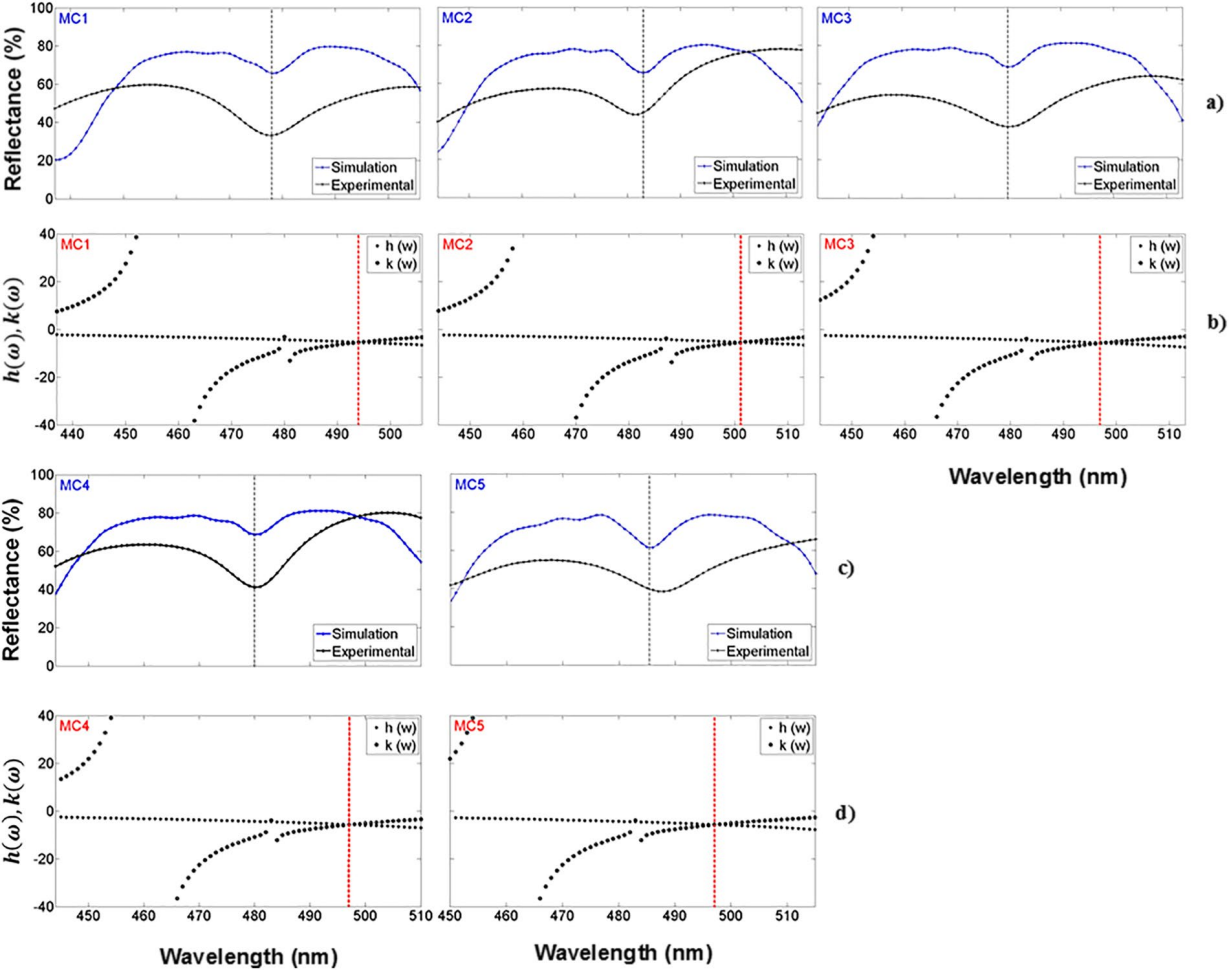
Comparison of the theoretical and experimental localized mode location in the VIS range; (**a,c**) show the theoretical (blue line) and experimental (black line) reflection spectrum of five MCs on Si substrates in the VIS range; (**b**,**d**) are the position of the theoretical localized mode of the same MCs calculated using the variational method.

A microcavity exhibits many photonic modes in its transmission spectrum; some of them are known as localized or localized modes and others as extended modes. The localized mode frequency always lies inside the PBG. In our work, the MCs were designed to present a localized mode within the UV-VIS range. The microcavity structure was antisymmetric and the Fig. 8b,d show the theoretical location of the localized mode of five MCs on a Si substrate in the VIS range. Their reflection spectrum is presented as well in Fig. 8a,c. The localized modes are shown as a reflection minimum at a specific wavelength between the PBG edges. A variational method along with the transfer matrix method were used to calculate the localized mode position^39^. The variational method localized mode location was 16 nm off with respect to the experimental result, while the transfer matrix method predicted the same position as the experimental result.

The wavelength of the localized mode depends on the value of the refractive index in the defect layer; the width of the peaks can be narrowed, and the steepness of the transmission curve increased by extending the length of the defect segment or by increasing the number of periods in the reflectors^39^.

**Figure 9:**
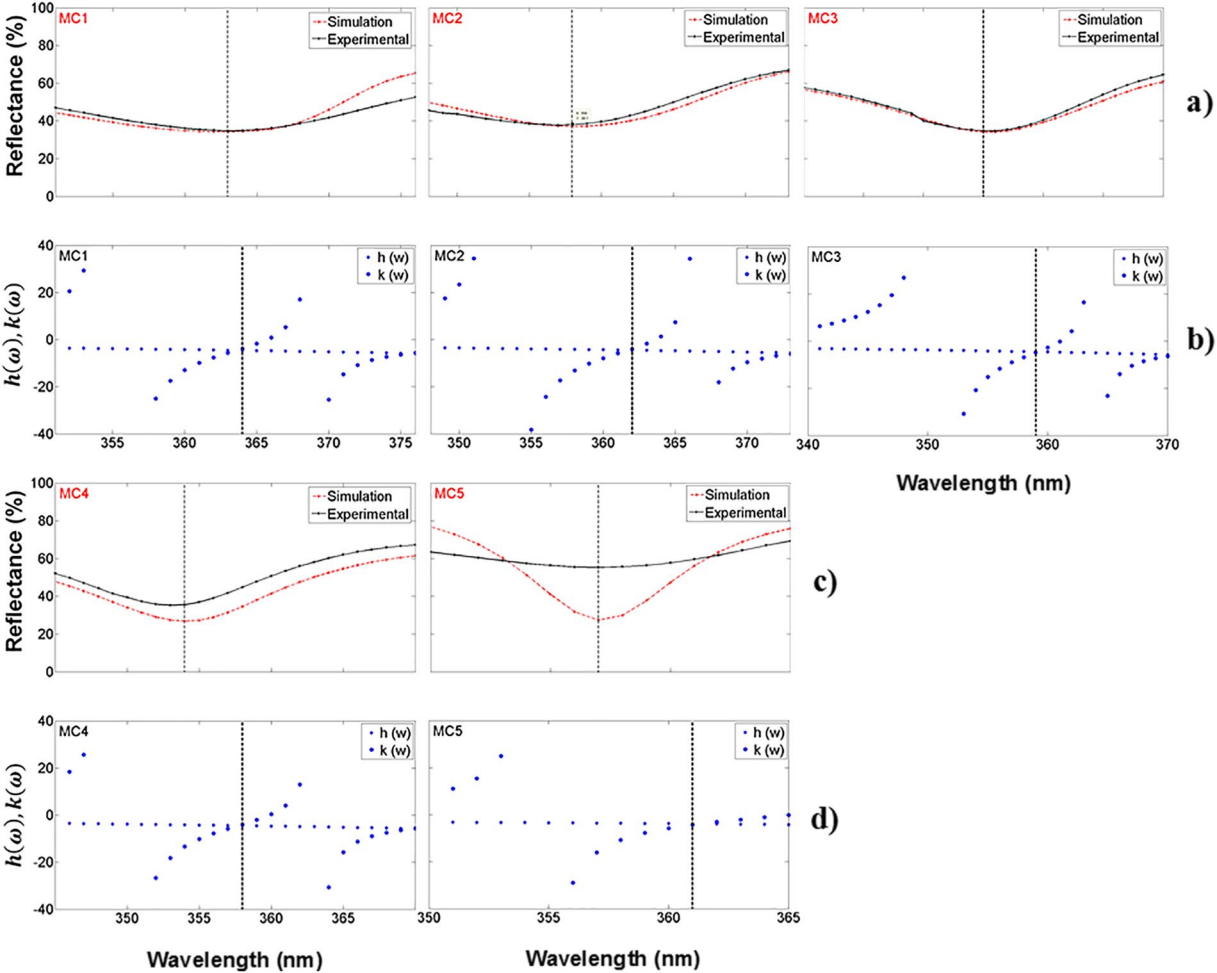
Comparison of the theoretical and experimental localized mode location in the UV range; (**a,c**) show the theoretical (red line) and experimental (black line) reflection spectrum of five MCs on Si substrates in the UV range; (**b,d**) are the position of the theoretical localized mode of the same MCs calculated using the variational method.

The comparison between the theoretical and experimental reflection spectrum within the UV range of five MCs on Si substrates is shown in Fig. 9a,c, and its theoretical localized mode location calculated with a variational method is exposed in Fig. 9b,d, inside the UV range. The wavelength position of the localized mode in the UV range was depicted in Table 4. It can be seen between the edges of the PBG as shown in Figs. 3–6. From Fig. 9 it can be observed that there is a good fit between the localized mode position predicted by the transfer matrix method and the experimental result, while the variational method predicted a localized mode position that was 3 nm off with respect to the position of the experimental localized mode.

**Figure 10:**
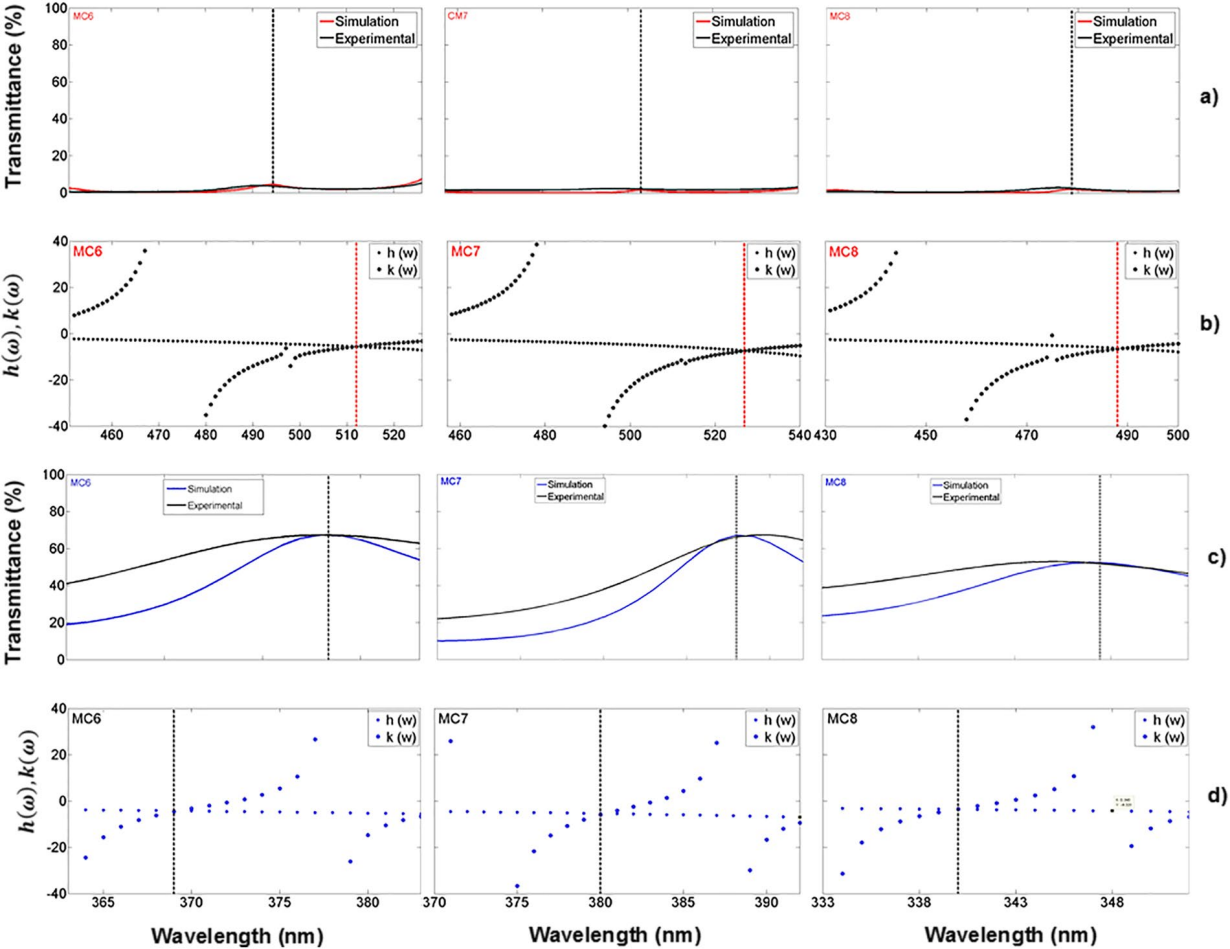
Comparison of the theoretical and experimental localized mode location of three MCs on quartz substrates, in the VIS and UV range; (**a**) shows the theoretical (red line) and experimental (black line) transmission spectrum in the VIS range; and (**c**) shows the theoretical (blue line) and experimental (black line) transmission spectrum in the UV range; (**b,d**) show the position of the theoretical localized mode of the same MCs, calculated using the variational method.

The localized modes are also found in the transmission spectrum. In Fig. 10a,c the transmission spectra of three MCs on a quartz substrate with their theoretical localized mode locations can be seen, as calculated using the variational method, which are displayed in Fig. 10b,d. In the VIS range, the MCs transmission spectrum shows a shallow peak at the location of the localized mode mainly due to photon losses probably caused by light absorption, while the MCs transmission spectrum in the UV range presented a higher amplitude peak at the localized mode location and therefore a smaller photon loss by light absorption.

All the theoretical localized modes shown in Fig. 10b,d, are confined within the PBG bandwidth; they are displaced with respect to the experimental location while the transfer matrix method predicted almost the same experimental localized mode location. The mislocation between the theoretical and experimental localized mode was 20 nm in the VIS region, and 8 nm in the UV region.

The localized modes are used for sensor applications. Photonic structures presenting localized modes have been exposed to various solvents as alcohols (ethanol, isopropanol, methanol), wines, deionized water; these compounds affect the defect region by changing its refractive index due to the absorption of molecules inside the pores. Commonly, the localized mode peaks shift toward low-frequencies (long wavelengths) because the solvents used have a higher refractive index value than that of air.

### Photon losses analysis of microcavities

**Figure 11:**
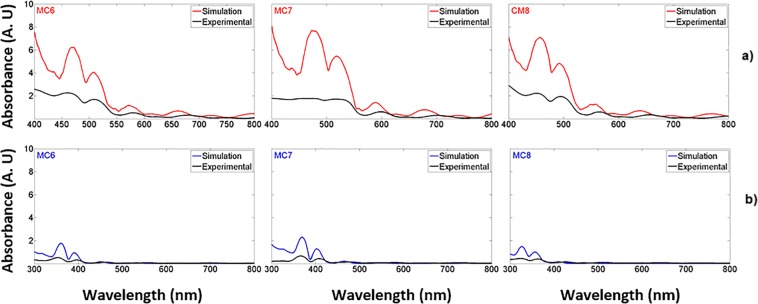
Theoretical and experimental absorbance spectra comparison for three MCs on quartz substrates. (**a**) Theoretical (red line) and experimental (black line) absorbance spectra in the VIS range and (**b**) theoretical (blue line) and experimental (black line) absorbance spectra of the same three MCs in the UV range.

MCs centered in the infrared and the visible red region have been reported before, where the PS is almost transparent. Absorption losses play a much less significant role than other loss mechanisms such as light scattering or dispersion by the pores and the interfaces between layers^12,24^; however, in the UV-VIS range both loss mechanisms have to be considered. We studied the absorption and Rayleigh scattering losses in UV microcavities. First, both the theoretical and experimental absorbance spectrum in the UV-VIS range was calculated using the Beer-Lambert law. Figure 11 depicts the absorbance spectrum of three MCs on quartz substrates. The red line is the theoretical absorbance, and the black line is the experimental absorbance for MCs in the VIS region (Fig. 11a). The MCs absorbance spectra in the UV region are shown in Fig. 11b; the theoretical absorbance spectrum is represented by the blue graph, while the experimental absorbance is presented by the black graph; both theoretical and experimental absorbance spectra have the same order of magnitude. It can be clearly seen that the absorbance decreases more in the OM due to the presence of silicon dioxide (SiO_2_) inside of PS layers.

**Figure 12:**
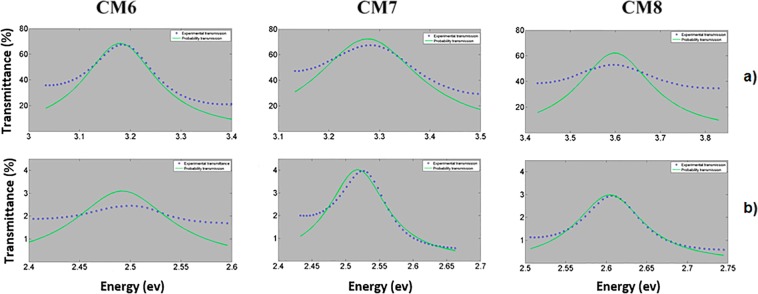
Comparison of the experimental transmission spectrum (blue line) with the transmission calculated by means of the Breit-Wigner´s equation modified by Miller (green line) for three MCs on quartz substrates. (**a**) the UV range and (**b**) the VIS range.

Second, a theoretical analysis of absorption and Rayleigh scattering losses, at the localized mode wavelength have been realized in this work. We used the equation of Breit-Wigner modified by Miller to get the lifetime of photons and photon losses at the localized mode wavelenght^25,30^. The modified Breit-Wigner equation is used to fit the experimental transmission spectrum (blue line) as a function of energy. The oxidation process modifies the localized mode position and also sharpens the microcavity resonance bandwidth as can be observed in Fig. 12. Table 6 shows the lifetime of photons and photon losses at the localized mode wavelength in the VIS and UV range of three MCs.

**Table 6:** The lifetime of photons in the VIS and UV range.

Microcavity	The lifetime of photons in the VIS (ps)	The lifetime of photons in the UV (ps)	Photon losses in the VIS (cm^−1^)	Photon losses in the UV (cm^−1^)
MC6	0.016	0.0355	2083	939
MC7	0.014	0.0434	2381	768
MC8	0.016	0.031	2083	1075

We found that the lifetime of photons in the VIS range is smaller than the lifetime of photons in the UV range as shown in Table 6. This shows that the photons losses in the UV range decreases more than 50% when compared with the VIS range losses.

How much of these losses are due to Rayleigh scattering only? And, is the level of Rayleigh scattering higher, in the visible or UV range? These are relevant questions because the scattering should become more pronounced in the UV range, as the ratio between the size of the porous structure and the wavelength increases. Thereby this scattering may limit the ultimate performance of the UV components that are made with the proposed approach.

We can estimate photon losses due to Rayleigh scattering before and after oxidation in our samples. We used a quantum mechanical model of scattering that takes into account the porous structure disorder, which allows the estimation of the total rate of Rayleigh scattering from the fundamental microcavity mode^25^. This model treats the features of porous silicon structure as a conglomeration of crystalline silicon wires with typical radius *a*_⊥_ and typical length *a*_||_, branches begin to have fluctuations and move away from a cylindrical shape. Due to these fluctuations in the dielectric constant, the Rayleigh scattering is an important parameter as a medium of energy loss. In this model, the parameters *a*_⊥||_ should be smaller than the period of the Bragg mirrors, that is *a*_⊥||_ < *d*_*H*_,*d*_*L*_ (see methods for details).

Estimating Rayleigh scattering in the UV range is straightforward. Since the fraction of crystalline silicon is less than 1% in samples MC6, MC7, and MC8), we can infer that all optical losses *α* in the UV range are due to Rayleigh scattering. After using the quantum mechanical model, in average Rayleigh scattering levels are lower in the UV range than the visible range in 20%. In average Rayleigh scattering contributes up to 55% in the visible range. All results are summarized in Tables 7 and 8.

**Table 7:** Parameters to obtain Rayleigh scattering losses in the UV range.

Microcavity	*p*	1 − *p*	*a*_⊥_(*m*)	*a*_||_(*m*)	〈(*δε*)^2^〉_*V*_(*m*^3^)	ε^*^	${{\boldsymbol{\omega }}}_{0}(\frac{1}{{\boldsymbol{s}}})$	${\boldsymbol{D}}({{\boldsymbol{\omega }}}_{0})(\frac{{\boldsymbol{s}}}{{{\boldsymbol{m}}}^{3}})$	${{\boldsymbol{\alpha }}}_{{\boldsymbol{RSL}}}(\frac{1}{{\boldsymbol{cm}}})$
MC6	0.10	0.9	5.5E-9	6.3E-08	2.3E-23	2.0	5.5E15	3.3E05	939
MC7	0.09	0.91	5.5E-9	9.0E-08	3.1E-23	2.1	4.8E15	2.6E05	768
MC8	0.13	0.87	5.5E-9	7.9E-08	3.7E-23	2.0	5E15	2.6E05	1075

**Table 8:** Parameters to obtain Rayleigh scattering losses in the visible range.

Microcavity	*p*	1 − *p*	*a*_⊥_(*m*)	*a*_||_(*m*)	〈(*δε*)^2^〉_*V*_(*m*^3^)	ε^*^	${{\boldsymbol{\omega }}}_{0}(\frac{1}{{\boldsymbol{s}}})$	${\boldsymbol{D}}({{\boldsymbol{\omega }}}_{0})(\frac{{\boldsymbol{s}}}{{{\boldsymbol{m}}}^{3}})$	${{\boldsymbol{\alpha }}}_{{\boldsymbol{RSL}}}(\frac{1}{{\boldsymbol{cm}}})$
MC6	0.46	0.54	2.5E-9	4.9E-08	1.5E-22	5.4	3.9E15	7.3E05	974
MC7	0.44	0.56	2.5E-9	7.1E-08	2.1E-22	5.8	3.9E15	8E05	1347
MC8	0.49	0.51	2.5E-9	6.3E-08	1.9E-22	5.3	3.9E15	6.8E05	1277

From these results, we can conclude that dry oxidation helps to reduce absorption and scattering losses in the UV region; forming SiO_2_ on the surface of the filaments throughout the porous structure, the oxide perfectly penetrates the pores; this is possible due to the particle size of oxygen (276 pm). The Si filaments thickens because part of Si is converted into SiO_2_ and the physical thickness of each PS layer grows. The thickness of SiO_2_ grown on PS filaments depends upon the temperature and oxidation time.

SiO_2_ is a transparent material at short and larger wavelengths; for this reason, the extinction coefficient is almost negligible. The refractive index change of oxidized PS structures is the result of the formation of SiO_2_ inside the Si matrix; the Si with refractive index around of 3.5 is consumed forming OPS and part of the air with refractive index equal to 1 is replaced by SiO_2_ reducing the pore size. The values for the refractive index of Si and SiO_2_ are reported in the literature^48^.

### Example of the use of porous Si-SiO_2_ microcavities within the UV region

**Figure 13:**
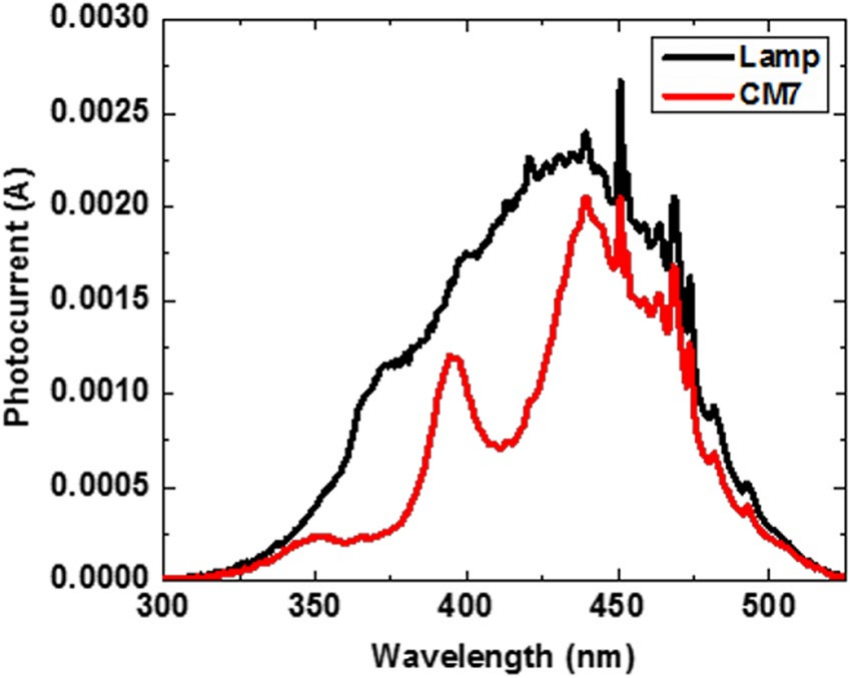
Photocurrents spectra of a commercial photodetector with and without porous Si- SiO_2_ microcavity filter. The black line represents the photocurrent spectra of a commercial photodetector GVGR-T10GD based in Indium Gallium Nitride with a spectral detection range from 300 to 510 nm (black line). The red line illustrates the modulated photocurrent with a porous Si-SiO_2_ microcavity filter. A Xenon lamp (6254) was used to measure the photocurrents.

One specific application is to use porous Si-SiO_2_ microcavities to modulate the responsivity of a broadband photodetector in the UV region. The advantage of this kind of application is that the photodetector will be more selective to some specific UV wavelengths due precisely to the narrowness of the photonic bandgap. An example of this application is shown in Fig. 13, where an UV microcavity (MC7) was used to modulate the photocurrent of a photodetector (black line). It was modified when an UV microcavity was placed on top of the photodetector (red plot).

The maximum transmission, due to the localized mode of sample MC7, dominates over other wavelengths within the UV range and induces a photocurrent maximum peak in the UV-A range. In this way, the photo-response of a photodetector in the UV was narrowed by using a porous Si- SiO_2_ microcavity filter.

## Conclusions

We designed and fabricated free-standing membrane microcavities (MCs) on quartz substrates and MCs on Si substrates in the UV range, all MCs were manufactured initially using porous silicon^40^. Their photonic band structures were measured employing reflection and transmission spectroscopies and theoretically using the matrix transfer method, and the agreement between experiment and theory was quite good. From the experimental reflection and transmission spectra, a localized mode can be observed within the PBG; and from SEM the spatial presence of a defect layer is clearly noticeable. We theoretically confirmed the very nature of such mode and indeed they are localized modes. The dry oxidation is used to obtain MCs in the UV band. The presence of silicon dioxide was confirmed by FTIR absorbance spectra. The characteristic bands with main peaks at 795, and 1015  cm^−1^ correspond to the bending, and stretching, of Si-O-Si bonds, respectively. The position and the shape of the main Si-O-Si vibrational band at 1015 cm^−1^ might indicate a stoichiometric composition^49,50^. Moreover, a carbon impurity vibrational band is also observed in the FTIR spectra, whose peak is smaller than the main Si-O-Si peak. The SiO_2_ grows within the porous structure. The excellent quality of silicon dioxide grown on the structure modifies the optical path and porosity. Thus, the refractive index and photon losses decreased, and the localized mode wavelength position shifted towards lower wavelengths.

Additionally, the transmission and reflection spectra showed a maximum and minimum peak respectively at the localized mode wavelength, for both VIS and UV bands. Besides the shifts, we found an increase in the transmission spectrum amplitudes at the localized mode wavelength of MCs in the UV range when compared with the one found in the VIS range. The change of the refractive index was known by using a three-component effective medium approximation model. Three MCs presented a transmission maximum of 5% in the blue range and 67% in the UV at the localized mode wavelength. The reflection minimum at the localized mode wavelength of five MCs on Si substrates was approximately 40% on the blue and UV range and then it had a value of 55% for one OM (oxidation time of 120 minutes).

Furthermore, the two-stage dry oxidation process presented here has proven to be very effective for obtaining complex photonic structures such as MCs in the UV region. The importance of the pre-oxidation stage in the strategy of thermal growth of high grade oxides on Si was made evident by the high-quality OMs shown by SEM, where the layer’s mechanical stability was preserved after the second oxidation stage at high temperature. This is quite impressive because, let’s not forget, that MC layers initially made of PS were transformed almost completely in SiO_2_ layers.

Notwithstanding, one might think that the refractive index contrast of the porous SiO_2_ is much limited compared to porous silicon. The reported effective refractive index for the porous SiO_2_ is only from 1.35 to 1.59. The small index contrast may limit the achievable performance of the optical components, as indicated by the small photonic bandgap (only ~20 nm) of the demonstrated microcavities.

The need for broad or narrow refractive index contrast would depend on the type of application is desired. For instance, if we think in sensor applications or light-emitting devices, a significant refractive index contrast is not necessary. Many sensing techniques using photonic devices utilize localized photonic modes^51^. Nonetheless, if a significant refractive index contrast is needed, some experimental approaches could be implemented with the intention of increase such difference.

First, it is known that as the ratio n_H_/n_L_ increases, the photonic bandgap increases. So, the refractive index contrast of porous Si- SiO_2_ could be enlarged because our microcavity has initially been manufactured from porous silicon. Porous silicon can have a refractive index contrast with values close to 1 up to 3.5. Consequently, after oxidation, the refractive index contrast ranges from values close to 1 up to 1.59. Indeed, in this work, the porous silicon refractive index contrasts were small, and that is why we obtained slight index contrast for porous Si-SiO_2_.

Second, to overcome the small refractive index contrast limitation, some authors have proposed a new method to expand the photonic bandgap in porous silicon structures introducing Bragg reflectors and coupled multiple microcavities designed at different wavelengths from VIS to infrared^20^. In this way, the photonic bandgaps of each porous silicon structure overlap, achieving a broader photonic bandgap. The same method could be applied to porous Si-SiO_2_ structures in the UV range. Notably, one way to obtain a broader optical response from these kinds of photonic structures is using chirping techniques^52^.

Third, small refractive index contrasts are found in some other photonic structures. For example, in photonic crystal fibers. Such fibers are made from undoped fused silica^53^. The core region of the fiber is formed by silica with a higher index (n_Hcore_ = 1.45) than the average index of the cladding. The cladding of photonic crystal fibers consists of a large number of air-holes embedded within a silica background. The region of pure silica forms a waveguiding core. Waveguiding occurs because the “holey“ fiber cladding effectively has a lower refractive index (n_Lcl_ = 1) than the pure silica core, resulting in total internal reflection at the core-cladding interface^54^. Therefore, the light is confined to the central core by reflection from the cladding that surrounds it^55^. All conventional fibers guide light by total internal reflection (TIR), which requires that core have a higher refractive index than the cladding. Despite small index contrast, these technologies have been proven to work in the telecommunications industry.

Four, we have given a specific example on how to benefit from the small index contrast of the porous Si-SiO_2_ microcavity to narrow down the responsivity of a broadband photodetector in the UV region. The filtering process was successful and in Fig. 13 we observed how the photodetector responses became more selective to some specific UV wavelengths. All these points prove that the refractive index contrast does not limit the achievable performance of optical components.

Another important point to discuss is what is the advantage of our approach compared to physical vapor deposition (PVD)? Because one can use PVD to deposit materials that have high refractive indices in the UV range, such as Hafnium dioxide (HfO_2_) with refractive index of n ~ 2.25 and Zirconium dioxide (ZrO_2_) that has a refractive index of n ~ 2.35, as well as low-index materials, such as SiO_2_ (n ~ 1.57).

The advantages of our approach compared to physical vapor deposition are among others; the manufacturing time is short, the cost is low, and the samples are easy to fabricate. On the contrary, physical vapor deposition requires temperatures ranging from 52 °C to 750 °C and pressures in the range from 1 × 10^−8^ Torr to 5.3 × 10^−2^ Torr to produce HfO_2_, ZrO_2_ and SiO_2_ layers on silicon, quartz, and sapphire^56–58^ substrates. The target used in this case is made of hafnium and zirconium (purity 99.99%)^40,56,57^ with a diameter base between 13 and 100 mm. The deposition time to obtain HfO_2_ and ZrO_2_ layer is 1 nm/min^59^, which is very long compared with our fabrication process. Besides, physical vapor deposition infrastructure is expensive.

If a microcavity of 1.6 µm thickness could be manufactured by physical vapor deposition, then the deposition process to form the structure will take more than 24 hours, while in our manufacturing process it takes 3.5 minutes to create the microcavity and 2 hours more to oxidize the microcavities.

Recently, it has been reported the fabrication of Bragg reflectors based on a mixture of different materials (HfO_2_ + ZrO_2_/ SiO_2_). The multilayer structure had 11 periods, where a maximum reflectance peak of almost 100% has been observed at 355 nm^60^. A mixture of HfO_2_ + ZrO_2_ was used as a high refractive index layer, and SiO_2_ was used as a low refractive index, and the refractive index contrast is a little bit broader than the reported in our work. However, the deposition of these materials was done by plasma ion-assisted deposition, which is a costly and slow technique. HfO_2_ and ZrO_2_ absorb light in the UV range^40,57^, where the imaginary part (extinction coefficient) of the refractive index is not negligible, it is a limiter to manufacture photonic structures of good optical transparency quality. That is why the use of mixtures is preferred because of a possibly better UV transparency compared to pure hafnia layers^60^. Another point to consider while using these high dielectric oxide films is they do not show sufficient thermal stability because the structure of the oxide films is easily converted from amorphous to polycrystalline and react with the Si substrates^61^.

In our opinion, SiO_2_ layers are the key to achieve photonic structures in the UV range, since it is not an absorbent material there, thus is considered a transparent material in that part of the electromagnetic spectrum. In this work, it was possible to achieve porous Si-SiO_2_ structures more complicated than just a Bragg reflector like the one based on HfO_2_ + ZrO_2_/ SiO_2_. Our structure has a localized mode, which shows a transmission maximum of 70% in the UV range, a new result to the best of our knowledge.

It is interesting to observe that Rayleigh scattering losses before oxidation are higher than Rayleigh scattering losses after oxidation. This result implies that the oxidation process not only helps diminish absorption losses but scattering losses as well. The reason why Rayleigh scattering is less after the oxidation is mainly due that the arithmetic mean of the volume-averaged fluctuation of porous silicon dioxide dielectric constant is 1/6 of its porous silicon counterparts; the same goes for the density of states whose mean value decreases 2/5 (See methods). Now the other two factors in Eq. 19 (methods) that increase after oxidation are ${\omega }_{0}^{2}$ and ε^*−2^, the former increases by a factor of 1.7, and the latter contributes to an increase of 7.3. Therefore, on average we should expect that Rayleigh scattering losses after oxidation will decrease by a factor of $1\,-\,1.7x7.3x2/30\,\approx \,0.20$. Even if we consider absorption losses are not zero in the UV range, the same result holds, that is there are more Rayleigh scattering losses in the visible range than in the UV.

In summary, scattering should indeed become more pronounced in the UV range, as the ratio between the size of the porous structure and the wavelength increases but only when there is not a phase change which it is not the case here because a porous structure based on crystalline silicon is transformed almost entirely into a porous structure based on silicon dioxide.

Moreover, the use of p^+^ substrates also helps out reducing scattering losses. In reference^24^ it has been reported that scattering depends strongly, first, on the layer thickness (size of the porous structure) and secondly on the doping level of the substrate, during the formation of porous silicon, porous silicon layers develop a roughness which is responsible for the observed scattering light, scattering in the interface between PS and air has been found negligible (<1%) compared to the interface of the volume of the porous silicon layer on p-type substrate, however using p^+^ substrate the scattering light level is lower. Besides, the volume scattering loss can always be reduced by applying thermal oxidation, where AFM measurements shown a roughness decrement after oxidation, the thermal oxidation has a smoothening effect on porous silicon layers^41^.

In the future, it would be possible by using this oxidation process the making of other kind of photonic structures like waveguides, Fibonacci filters, multi-cavities, and rugate filters. Among other possible applications, for instance, the UV microcavities can be used to modulate the optical response of photodetectors such as gallium nitride (GaN) and zinc oxide (ZnO) to achieve a much more selective photoresponse in the UV. Also, it can be used as vibration sensor due to the known SiO_2_ piezoelectric properties. This procedure expands the field of research of silicon based photonic structures in the UV range.

## Methods

### Electrochemical anodization of microcavities

The porous silicon MCs were obtained by electrochemical anodization on highly doped p^+^ type $(\rho \,=\,0.01\,-\,0.02\,\,\Omega cm)$ crystalline silicon (c-Si) wafers, (100) orientation; before the electrochemical anodization the c-Si wafer was immersed in a solution of 20% HF for 5 minutes to remove native oxide; after, it was rinsed with deionized water and vertical positioned to let it dry at room temperature. The c-Si wafer with dimensions 1.5 *cm* × 1.5 *cm* was put in a Teflon cell with etching area of 1 cm² that was used to carry out the electrochemical process. Then an aqueous electrolyte of 40% HF and ethanol at 99.7% with a volume ratio of 1:1 was placed in the Teflon cell. A ring-shaped tungsten electrode immersed in the electrolyte was used as the cathode, and an aluminum plate that contacts the unpolished backside of the c-Si wafer was used as the anode.

A power supply (Keithley 2460) controlled by a laptop was used to deliver the current profile of MCs. The current profile consisted in interchanging two different current pulses, the first current pulse is 5 mA/cm^2^ (low porosity) for 4.1 seconds (s), the second current pulse is 80 mA/cm^2^ (high porosity) for 1.1 seconds (s) and finally a current pulse of 80 mA/cm^2^ (high porosity) for 2.2 seconds is used to form a defect layer. After each current pulse, a pause of 3 seconds was introduced to generate the flow of the electrolyte and prevent porosity gradients. The defect is an essential characteristic of MCs, which is created between two BRs and ours present a localized photonic mode.

### Self-supported microcavity

The microcavity was self-supported on a quartz substrate with dimensions of 1.6 cm × 1.6 cm. The MC lifting up was carried out with an electrolyte of 40% HF and ethanol at 99.7% with a volume ratio of 1:1 in a Teflon cell, and a high current pulse of 450 mA/cm^2^ was applied for 2 s to the c-Si substrate. When the manufacturing process finished, and the MCs were self-supported, they were rinsed with ethanol and dried in the environment.

### Dry oxidation

The obtained MCs were subjected to two stages of dry oxidation; the first stage was a low-temperature pre-oxidation at 350 °C for 30 minutes that prevents PS layers collapsing during an additional heat treatment at high temperatures^16^.

The second stage was a high-temperature oxidation at 900 °C, applied to grow an oxide layer of greater thickness than the obtained in the preceding oxidation stage; the layer thickness is higher than the natural native oxide grown in the environment, thus leading to the consolidation of SiO_2_. At this stage the oxidation temperature was constant, and the oxidation time ranged from 30 up to 120 minutes. The samples were removed from the oxidation system when it reached room temperature.

### Optical and structure characterization

The optical characterization of MCs was carried out with a Varian (Agilent) UV-Vis-NIR spectrophotometer at normal incidence and an incident angle to 20 degrees, and FTIR measurements (Varian 660 IR) were performed in attenuated total reflection mode in the spectral range 390–4000 *cm*^−1^. Images of Scanning Electron Microscopy (JEOL JSM7600F) were obtained to examine the geometrical characteristics of the MCs. All MCs were measured before and after dry oxidation. Characterization of the MCs was performed by SEM, FTIR and UV-Vis-NIR spectroscopy before and after the dry oxidation.

### A theoretical model for calculating the refractive index

The effective medium theory was used to get the refractive index of two (Si, air) and three mediums (SiO_2_, Si, and air). The literature discusses different effective medium theory approximations such as those developed by Looyenga, Maxwell-Garnett, and Bruggeman, to obtain the refractive index of PS layers^4,31,62^.

In this case, we considered the Maxwell-Garnett’s equation; this equation was used to calculate the theoretical refractive index value of PS, where it is considered as a homogeneous medium with an effective complex dielectric function. The equation for two mediums is expressed as follows:

1 $$\frac{{\varepsilon }_{PS}-{\varepsilon }_{Si}}{{\varepsilon }_{PS}+{\varepsilon }_{Si}}=P\frac{{\varepsilon }_{air}-{\varepsilon }_{Si}}{{\varepsilon }_{air}+{\varepsilon }_{Si}}$$

where *P* (porosity) is the air fraction of the non-oxidized PS layers, *ε*_*air*_ is the dielectric constant of air; *ε*_*Si*_ is the dielectric constant of Si, and *ε*_*PS*_ represents the effective dielectric constant of PS.

The simplest model proposed by J. E. Lugo was used to obtain the refractive index of OPS, which takes into account three components: Si, SiO_2_, and air. This model is an extension of Maxwell-Garnett; the J. E. Lugo model considers the presence of SiO_2_ and its network expansion that occurs within the porous structure, due to the increase of SiO_2_ in the porous matrix of SP^31^.

The refractive index for three components can be obtained using the following equation:

2 $$\frac{{\varepsilon }_{PS}-{\varepsilon }_{Si}}{{\varepsilon }_{PS}+{\varepsilon }_{Si}}={P}^{\ast }\frac{{\varepsilon }_{1}^{\ast }-{\varepsilon }_{3}^{\ast }}{{\varepsilon }_{1}^{\ast }+{\varepsilon }_{3}^{\ast }}$$

where ${\varepsilon }_{1}^{\ast }$ and ${\varepsilon }_{3}^{\ast }$ are given by

3 $${\varepsilon }_{1}^{\ast }\,=\,{\varepsilon }_{air}[1\,+\,\frac{{\varepsilon }_{{{\rm{SiO}}}_{2}}}{{\varepsilon }_{air}}\gamma ]$$

4 $${\varepsilon }_{3}^{\ast }\,=\,{\varepsilon }_{{{\rm{SiO}}}_{2}}[1\,+\,\frac{{\varepsilon }_{air}}{{\varepsilon }_{{{\rm{SiO}}}_{2}}}\gamma ]$$

where $\gamma \,=\,\frac{1\,-\,\beta }{1\,+\,\beta }$ and ${\varepsilon }_{{{\rm{SiO}}}_{2}}$ is the dielectric constant of SiO_2_.

The parameter β is represented by:

5 $$\beta \,=\,{[\frac{1-0.55x}{1+0.45x}]}^{2},$$

where *x* is a dimensionless oxidation parameter, ${P}^{\ast }=\frac{{P}_{ox}}{\beta }$ and *P*_*ox*_ is the porosity after the dry oxidation.

The porosity for a system of three components can be calculated as

6 $${P}_{ox}\,=\,P{[1-0.55x]}^{2}.$$

The oxidation parameter upper limit value, *x*_*L*_, that a given layer of PS can have with a specific porosity can be found solving the following expression:

7 $$P\ast (1+0.9\ast {x}_{L}+0.2015\ast {x}_{L}^{2})\,\le \,1.$$

The constants 0.55 and 0.45 in Eq. (5) are related to oxide growth in Si. Therefore, an oxide layer grows 55% above of the Si wafer surface and 45% below the original surface. Hence the Si is consumed as the oxide grows and a volume expansion occurs during oxidation^44^. We can obtain the oxide fraction (*f*_*ox*_) after dry oxidation as

8 $${f}_{ox}\,=\,P[2x\,-\,0.1{x}^{2}].$$

Therefore, the Si fraction *f*_*Si*_ can be calculated using the following equation:

9 $${f}_{Si}\,=\,1\,-\,{P}_{ox}\,-\,{f}_{ox}.$$

The effective medium theory for three components of Bruggeman and Looyegan also can be applied to find the refractive index of OPS. The comparison between the theoretical spectra of transmission and reflection of these three models were shown in the section of results and discussion.

### The Transfer-Matrix Method

The Transfer Matrix Method is used to calculate the theoretical transmission and reflection spectra for MCs. The transfer matrix is very well-known^38^; it can be resolved for N numbers of layers, and can be written as

10 $$(\begin{array}{c}{M}_{11}\,\\ {M}_{21}\end{array}\begin{array}{c}{M}_{12}\\ \,{M}_{22}\end{array})\,=\,{D}_{0}^{-1}[{\prod }_{l\,=\,1}^{N}{D}_{l}{P}_{l}{D}_{l}^{-1}]{D}_{s}$$

where *M*_11_, *M*_12_, *M*_21_, *M*_22_ are matrix elements; they are fundamental to describe how electromagnetic radiation propagates along a periodic medium; *D*_*l*_ is the dynamical matrix of PS, *D*_0_ is the dynamical matrix of air and *D*_*s*_ is the dynamical matrix of the substrate and *P*_*l*_ is the propagation matrix.

The equations to get the transmission and reflection are given by

11 $$T=\frac{{n}_{s}\,\cos \,{\theta }_{s}}{{n}_{0}\,\cos \,{\theta }_{0}}{|\frac{1}{{M}_{11}}|}^{2}$$

12 $$R={|\frac{{M}_{21}}{{M}_{11}}|}^{2}$$

where *T* represents the transmission, *n*_*s*_ is the refractive index of the substrate, *n*_0_ is the refractive index for air, *θ*_*s*_ is the output angle in the substrate, *θ*_0_ is the angle of incidence, and *R* is the reflection^38^.

We used the dispersion relation to obtain the theoretical photonic band structure of MCs; it can be written as

13 $$K(\beta ,\omega )\,=\,\frac{1}{d}{\cos }^{-1}[\frac{1}{2}({M}_{11}\,+\,{M}_{22})]$$

where *K* is the Bloch wave number, *β* is the off-axis wave vector component, *d* is the spatial period that equals the addition of individual thicknesses, and *ω* is the frequency. Indeed, the wave propagation in a periodic medium is very similar to the motion of electrons in crystalline solids^31,38^. The formation of forbidden bands where the propagation of photons is not allowed is produced by the periodic variation of PS dielectric constants. The most common example of the PBG realization is known as a BRs. A microcavity has one or more photonic modes where the photons can be localized in the defect region at some frequency and energy. Photonic modes are known as defect or localized modes and extended modes^39^; the first ones are located within the PBG band edges, while the second ones are located outside of the PBG edges and they are different from a localized mode, because their associated electromagnetic fields can propagate throughout the periodic medium. We have taken the absolute value of the refractive index and its wavelength average within the whole experimental spectral range for PBG structure calculations; it was necessary because this theory is not applicable to materials that present wavelength dispersion.

In order to find the theoretical frequency of a localized mode inside the bandgap, we use the method of the Optical Transfer Matrix (OTM)^39^. It is closely related to the Transfer Matrix Method, and it directly allows the description of the connection between adjacent regions by continuity conditions at the endpoints. It can be calculated at separate regions, so the optical properties of BRs with N periods and a defect layer are determined by considering a superposition of a right-traveling plane wave and a left-traveling plane wave.

Thus, the function ${\gamma }_{N}\,=\,\frac{{M}_{11}\,-\,{M}_{21}}{\,{M}_{22}\,-\,{M}_{12}}$ relates the matrix elements. It has a relationship to the function *κ*(*ω*), which depends on the frequency; and it can be written as:

14 $$\kappa (\omega )\,=\,{k}_{d}Im(\frac{1\,+\,{\gamma }_{N}}{1\,-\,{\gamma }_{N}}),$$

where *k*_*d*_ is the z component of the wavevector for the first layer (defect layer), where z-axe is taken along of the normal direction to the layers (Fig. 1). We can obtain the variation of the localized mode frequencies for an antisymmetric mode or a symmetric mode. In the antisymmetric case these frequencies can be expressed as:

15 $$h(\omega )\,=\,-\,{k}_{d}\,\cot ({k}_{d}L)\,{\rm{for}}\,{\rm{antisymmetric}}\,{\rm{modes}}$$

here *L* is the half-length of the defect layer and *k*_*d*_ is the z component of the wavevector as it was mentioned before. We consider only the antisymmetric case because our microcavity design is antisymmetric and the condition where the functions *κ*(*ω*) and *h*(*ω*) intersect gives the localized mode frequency. For the localized mode calculation, we used the wavelength average of the complex refractive index absolute value.

### Photon losses analysis of microcavities

The photon losses in the infrared range are mainly related to Rayleigh scattering on the rough structure of PS and in the VIS range due to absorption and scattering. One theoretical study on the optical losses at localized mode wavelengths of free-standing MCs has been reported. The localized mode is shown as one resonant transmission peak, whose amplitude is modified by optical losses. The equation of Breit-Wigner was modified by Miller to get the dispersion in the localized mode due to absorption and scattering losses^25,30^; it can be related to the transmission spectrum close to the localized mode wavelength. This equation is written as

16 $${\rm{T}}(\omega )=\frac{{\Gamma }^{2}}{{(\Gamma +{\Gamma }_{p})}^{2}\,+\,{(\omega -{\omega }_{0})}^{2}}$$

where Γ_*p*_ represents the photon loss rate in the microcavity whereby the lifetime of photons can be defined at the localized mode wavelength as *τ* = 1/Γ_*p*_, which also includes the Rayleigh scattering losses and absorption losses, which are given by *α* = 1/*τc*, where c is the speed of light.

The tunneling of photons in MCs follow Bose-Einstein statistics, where some of the photons can be scattered and lost; it is similar to the electron tunneling. Photon tunneling through in a microcavity goes from the first BR to the defect region and finally to the second BR.

We used the Beer-Lambert law to get the absorbance spectrum of MCs on quartz substrates; this equation can be expressed as

17 $$A\,=\,{\log }_{10}1/T$$

where A is the absorbance and *T* represents the transmission. Therefore, this equation is related to the light intensity that passes through the microcavity, where part of the light is absorbed, and part of the light is transmitted.

If the porosity is not very small or high, the volume-averaged fluctuation of porous silicon dielectric constant is well approximated by

18 $${\langle {(\delta \varepsilon )}^{2}\rangle }_{V}\,=\,16p(1-\,p){({\varepsilon }_{2}-{\varepsilon }_{1})}^{2}{a}_{\perp }^{2}{a}_{\parallel }$$

where *p* is the mean porosity of the porous structure, which takes values between 0 to 1. *ε*_1_ and *ε*_2_ are the minimum and maximum dielectric constant bounds of the porous and solid-phase regions, respectively. For PS *ε*_1_ = 1 and *ε*_1_ = 12 and porous SiO_2_ *ε*_1_ = 1 and *ε*_1_ = 3.9.

The Rayleigh Scattering Losses (RSL) can be obtained as

19 $${\alpha }_{RSL}=\frac{{\Gamma }_{RSL}}{c}\,=\,\frac{\pi {\langle {(\delta \varepsilon )}^{2}\rangle }_{V}{\omega }_{0}^{2}}{6{\varepsilon }^{\ast 2}c}D({\omega }_{0})$$

where Γ_*RSL*_ represents the Rayleigh Scattering Loss Rate in the microcavity, *ω*_0_ is the angular frequency of the localized mode, c is the speed of light and *D*(*ω*_0_) is the density of photon states in the Bragg Reflector given by $D({\omega }_{0})\,=\,{\varepsilon }^{\ast 3/2}{{\omega }_{0}}^{2}/{\pi }^{2}{c}^{3}$, which is close to the density of states in uniform media with the dielectric constant ε^*^ expressed as

20 $${\varepsilon }^{\ast }\,=\,\frac{{d}_{H}{\varepsilon }_{H}\,+\,{d}_{L}{\varepsilon }_{L}}{\Lambda }$$

where *d*_*H*_ is the high porosity layer thickness and *d*_*L*_ is the low porosity layer thickness, and Λ is the period (Λ = *d*_*H*_ + *d*_*L*_), *ε*_*H*_ and *ε*_*L*_ correspond to the dielectric constant of porous silicon layers, ${\varepsilon }_{H}\,=\,{n}_{H}^{2}$ for high porosity and ${\varepsilon }_{L}\,=\,{n}_{L}^{2}$ for low porosity.

It is known^63^ that $2{a}_{\perp }\,\approx \,5\,nm$ for the same type of crystalline silicon used here thus a value of 2.5 nm for *a*_⊥_ will be utilized before oxidation. The growth of an oxide layer of thickness *x*_0_ will consume a layer of silicon of 0.45*x*_0_ thick^31^. If all crystalline silicon becomes silicon dioxide, the final size for *a*_⊥_ will be $2.5\,nm/0.45=5.5\,nm$. The expansion of the radius occurs because the density of silicon dioxide is less than the density of crystalline silicon.

Since practically all optical losses *α* in the UV range are due to Rayleigh scattering (the fraction of crystalline silicon is less than 1% in samples MC6, MC7, and MC8) then *α* ≈ *α*_*RSL*_. Using the experimental values for *α* in the UV range, presented in Table 6, and feeding Eqs. 18 to 20 with the corresponding parameters values (see Table 7) we can estimate the typical length of the silicon dioxide wires ${a}_{\mathrm{||}Si{O}_{2}}$, whose related values are shown in Table 7. We can notice that their values are less than the period of the microcavities after oxidation.

Now we can use ${a}_{\mathrm{||}Si{O}_{2}}$ values to obtain *a*_||*Si*_ values by considering that after oxidation, the period sample increased by 27%. Table 8 shows the final values, and again their magnitudes are less than the samples periods before oxidation. Using these values and feeding Eqs. 18–20 again with the corresponding parameters values (see Table 8), we can estimate the related Rayleigh scattering losses before oxidation, whose values are displayed in Table 8.

The datasets generated during and/or analyzed during the current study are available from the corresponding author on reasonable request.
